# Serum-derived extracellular vesicles facilitate temozolomide resistance in glioblastoma through a HOTAIR-dependent mechanism

**DOI:** 10.1038/s41419-022-04699-8

**Published:** 2022-04-13

**Authors:** Xiaosong Wang, Xiaojun Yu, Haoran Xu, Kang Wei, Shanxi Wang, Yingguang Wang, Junfei Han

**Affiliations:** 1 Department of Neurosurgery, The First Hospital of Qiqihar, Qiqihar, 161005 P.R. China; 2 Department of Neurosurgery, Affiliated Qiqihar Hospital, Southern Medical University, Qiqihar, 161000 P.R. China; 3 Department of Orthopedics, Tongji Hospital, Tongji Medical College, Huazhong University of Science and Technology, Wuhan, 430030 P.R. China; 4 Department of Neurosurgery, Huizhou Third People’s Hospital, Guangzhou Medical University, Huizhou, 516002 P.R. China

**Keywords:** Neuroscience, Diseases

## Abstract

Extracellular vesicle (EV)-mediated transfer of long non-coding RNAs (lncRNAs) has been reported to regulate chemoresistance in various cancers. We herein investigate the therapeutic potential of bioinformatically identified HOTAIR transferred by serum-derived EVs (serum-EVs) in temozolomide (TMZ) resistance of glioblastoma (GBM) and the downstream mechanisms. EVs were isolated from the serum of GBM patients. Expression of HOTAIR was examined in the clinical tissue samples and serum-EVs of GBM patients. The downstream miRNAs of HOTAIR and its target genes were predicted in silico. The effects of the HOTAIR transmitted by serum-EVs in malignant phenotypes, tumor growth, and TMZ resistance were assessed in vitro and in vivo. HOTAIR expression was upregulated in clinical tissues, cells, and serum-EVs of GBM. Co-culture data showed that GBM-serum-EVs facilitated GBM cell proliferative and invasive phenotypes and TMZ resistance by elevating HOTAIR. In GBM cells, HOTAIR competitively bound to miR-526b-3p and weakened miR-526b-3p’s binding ability to EVA1, thus increasing the expression of EVA1. Furthermore, HOTAIR carried by serum-EVs promoted tumor growth and TMZ resistance in vivo by suppressing miR-526b-3p-mediated EVA1 inhibition. GBM-serum-EV-enclosed HOTAIR may augment GBM progression and chemoresistance through miR-526b-3p downregulation and EVA1 upregulation. These results provide a strategy to reduce TMZ resistance in GBM treatment.

## Introduction

Glioblastoma (GBM), one of the most lethal cancers, is the most frequently occurring primary tumor in the central nervous system in adults and leads to poor prognosis [1, 2]. Currently, standard treatment approaches for GBM are surgical methods, radiotherapies, and temozolomide (TMZ) chemotherapy [3]. Nevertheless, many GBM patients suffer from therapeutic resistance, leading to unfavorable tumor recurrence and poor survival [4]. As a result, knowledge of specific mechanistic basis underlying the TMZ resistance of GBM cells would allow for the active development of novel therapeutic targets.

The serum is an abundant and accessible source of extracellular vesicles (EVs) and serum-derived EVs (serum-EVs) have demonstrated diagnostic values in the central nervous system-related diseases by carrying a large diversity of molecules [5, 6]. EVs are membrane vesicles composed of exosomes and microvesicles, and EV-enclosed long non-coding RNAs (lncRNAs) are implicated in the malignancy of malignant tumors by regulating tumor cell proliferative phenotypes and chemoresistance [7]. LncRNA HOX transcript antisense RNA (HOTAIR) has been reported to be upregulated in TMZ-resistant GBM cells, and its poor expression represses the malignant features [8]. In addition, the deletion of HOTAIR element can alleviate glioma progression and chemoresistance to TMZ [9]. The starBase database analysis in our work predicted putative binding sites between HOTAIR and microRNA (miR)-526b-3p. miR-526b-3p is poorly expressed in glioma-associated endothelial cells and conversely, its upregulation impairs the viability and migration of glioma-associated endothelial cells [10]. In addition, a recent study also confirmed the downregulated miR-526b-3p level in both glioma tissues and cell lines, and that enforced miR-526b-3p can arrest glioma tumorigenesis and progression by targeting WEE1 [11]. miR-526b-3p binding sites in the 3′-untranslated region (3′-UTR) of epithelial V-like antigen 1 (EVA1) mRNA were predicted by the starBase database. EVA1 has been shown to induce the proliferative phenotypes of GBM cells, which are resistant to therapies, and lead to recurrence [12].

Hence, we hypothesized that serum-EVs may function in the GBM progression or chemoresistance through HOTAIR-dependent miR-526b-3p/EVA1 axis. To address this hypothesis, we probed the role of serum-EV HOTAIR in GBM progression and TMZ resistance and further investigated its potential molecular mechanism by subsequent bioinformatics analysis and a series of experiments.

## Results

### HOTAIR is highly expressed in clinical tissues, cells, and serum-EVs of GBM

**Fig. 1:**
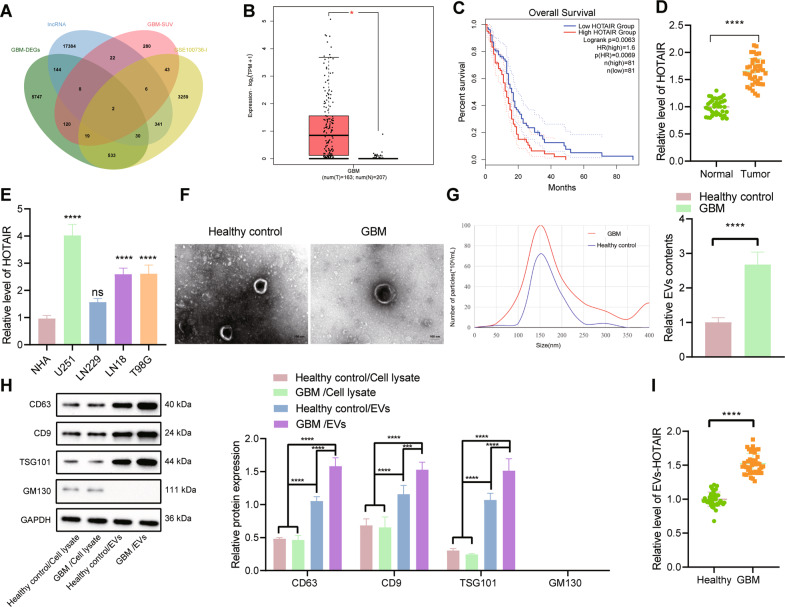
HOTAIR is upregulated in clinical tissues, cells, and serum-EVs of GBM. **A** Venn diagram of upregulated genes from the GEPIA database, the top 500 survival-related genes, upregulated lncRNAs from the GSE100736 data set, and human lncRNA names from the GENCODE database. **B** A box plot of HOTAIR expression in TCGA-GBM. The red box on the left indicates GBM samples, and the gray box on the right indicates normal samples. *p < 0.05. **C** Correlation analysis of HOTAIR expression with the overall survival of GBM patients in TCGA-GBM. **D** RT-qPCR detection of HOTAIR expression in normal brain tissues (*n* = 40) and GBM tissues (*n* = 40). ****p < 0.0001. **E** RT-qPCR detection of HOTAIR expression in GBM cell lines and human primary astrocytes NHA. ****p < 0.0001, ns, not significant, compared with NHA cells. **F** Morphology of serum-EVs of GBM patients (*n* = 40) and healthy controls (*n* = 40) observed under a TEM. **G** Graph (left panel) and quantification (right panel) of NTA results of the size distribution and relative concentration of serum-EVs of GBM patients (*n* = 40) and healthy controls (*n* = 40), ****p < 0.0001. **H** Western blot analysis of CD63, CD9, TSG101, and GM130 proteins in the serum-EVs of GBM patients (*n* = 40) and healthy controls (*n* = 40). **I** RT-qPCR detection of HOTAIR expression in serum-EVs of GBM patients (*n* = 40) and healthy controls (*n* = 40). ****p < 0.0001. Each experiment was repeated three times independently.

Analysis of the GEPIA database revealed 6603 significantly upregulated genes in GBM samples, and the top 500 survival-related genes were obtained following analysis on the relationship between GBM gene expression and survival. In addition, 1877 significantly upregulated lncRNAs were identified from the TMZ resistance-related GSE100736 data set, and the GENCODE database yielded 17,937 human lncRNA names. Following Venn diagram analysis of these lncRNAs, HOTAIR and HOXB-AS1 were found at the intersection (Fig. 1A). The significance of HOTAIR during survival analysis (*p* = 7.29e-3) was higher than that of HOXB-AS1 (*p* = 9.29e-3). Therefore, we chose HOTAIR for further research.

In the TCGA-GBM database, HOTAIR was found to be abundantly expressed in GBM and this upregulation was related to the poor prognosis of GBM patients (Fig. 1B, C). Quantitative reverse transcription PCR (RT-qPCR) test results showed that HOTAIR expression was augmented in clinical tissues of GBM patients (Fig. 1D). At the same time, HOTAIR expression was robustly induced in GBM cell lines U251, LN229, LN18, and T98G, of which U251 cells had the highest HOTAIR expression, and LN229 cells exhibited the lowest HOTAIR expression (Fig. 1E). These results indicate that HOTAIR is highly expressed in GBM.

TEM results showed that serum-EVs of GBM patients and healthy controls had similar morphological characteristics (Fig. 1F). NTA indicated that on the premise of EV isolation from the same volume of serum, the particle size distribution of EVs in the serum of GBM patients was similar to that of the healthy controls, but the peak value of EVs in the serum of GBM patients was much higher than that in the healthy controls (Fig. 1G, left panel). After quantification of the peak values of the NTA graph, it could be clearly seen that the number of EVs in the serum of GBM patients was significantly larger than that in the healthy controls (Fig. 1G, right panel). Moreover, western blot results suggested that compared with blood cell lysates, the high expression of EV markers CD63, CD9, and TSG101 could be detected in the serum-EVs of both GBM patients and healthy controls, whereas GBM-serum-EVs presented with higher levels of these markers following the same experimental procedures. Meanwhile, GM130 expression was not found in serum-EVs of both GBM patients and healthy controls. The results indicated the successful isolation of EVs and a larger number of EVs in serum of GBM patients (Fig. 1H). The detection results of RT-qPCR suggested significantly higher HOTAIR expression in serum-EVs of GBM patients compared with healthy controls (Fig. 1I). The aforementioned results indicate that HOTAIR is preferentially expressed in clinical tissues, cells, and serum-EVs of GBM.

### GBM-serum-EVs augment GBM cell malignant features and TMZ resistance by upregulating HOTAIR

**Fig. 2:**
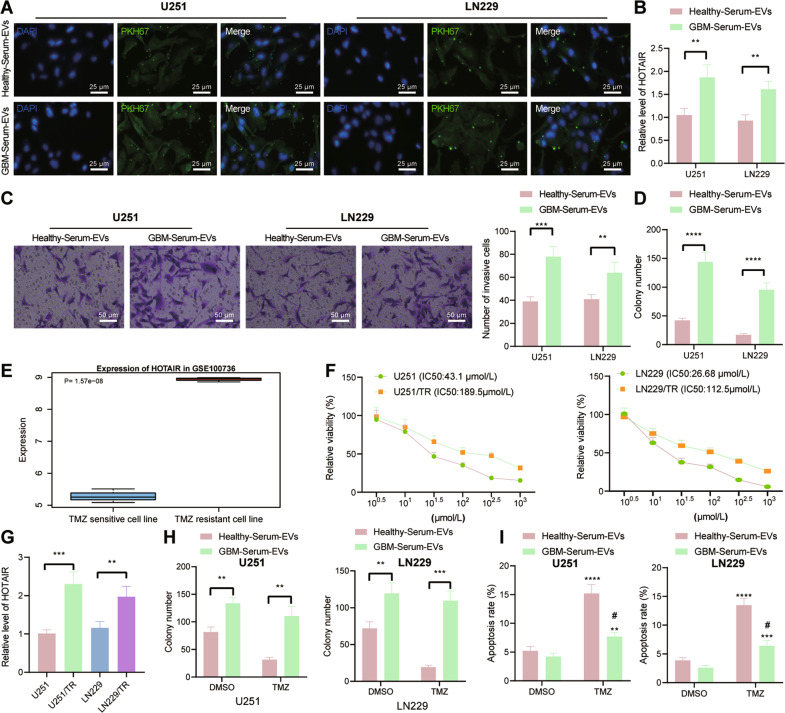
GBM-serum-EVs induce the malignant features as well as TMZ resistance of GBM cells by mediating HOTAIR. **A** Representative images of internalization of serum-EVs by U251 and LN229 cells under a fluorescence microscope. **B** RT-qPCR detection of HOTAIR expression in U251 and LN229 cells co-cultured with GBM-serum-EVs and healthy-serum-EVs. **p < 0.01. **C** Invasion of U251 and LN229 cells co-cultured with GBM-serum-EVs and healthy-serum-EVs measured by Transwell assay. **p < 0.01, ***p < 0.001. **D** Colony formation of U251 and LN229 cells co-cultured with GBM-serum-EVs and healthy-serum-EVs measured by colony formation assay. ****p < 0.0001. **E** HOTAIR expression in the GSE100736 data set. **F** IC50 of U251, U251/TR, LN229, and LN229/TR cells co-cultured with GBM-serum-EVs and healthy-serum-EVs measured by CCK-8. **G** RT-qPCR detection of HOTAIR expression in TMZ-resistant U251/TR and LN229/TR cells and its parental cells U251 and LN229. **p < 0.01, ***p < 0.001. **H** Colony formation of U251/TR and LN229/TR cells are co-cultured with GBM-serum-EVs and healthy-serum-EVs measured by colony formation assay. **p < 0.01, ***p < 0.001. **I** Flow cytometric analysis of apoptosis of U251/TR and LN229/TR cells co-cultured with GBM-serum-EVs and healthy-serum-EVs. ****p < 0.0001, compared with cells treated with DMSO. ^#^*p* < 0.05, compared with healthy-serum-EVs. Each experiment was repeated three times independently.

Next, we shifted our attention to determine the effect of serum-EVs on GBM cell function and resistance of GBM cells to TMZ by mediating HOTAIR. To exclude the effects of the difference between the serum-EV content of GBM patients and healthy controls, we used the same number of the two kinds of serum-EVs for subsequent experiments. Under a fluorescence microscope, fluorescence distribution was obvious in the U251 and LN229 cells co-cultured with GBM-serum-EVs labeled with the green fluorescent dye PKH67 for 24 h (Fig. 2A), which indicated effective internalization of the serum-EVs, either from GBM patients or healthy controls, by GBM cells. Higher expression of HOTAIR was witnessed in U251 and LN229 cells in response to co-culture with GBM-serum-EVs than with healthy-serum-EVs (Fig. 2B). The results of Transwell and colony formation assays displayed an increase in the proliferative and invasive phenotypes of GBM-serum-EVs-treated U251 and LN229 cells (Fig. 2C, D).

To construct TMZ-resistant GBM cells, the original TMZ-sensitive U251 and LN229 cells were exposed to TMZ of gradually increasing concentration (to a final concentration of 400 μmol/L) during 6-month incubation, and 50 μmol/L of TMZ was kept in the medium for the obtained U251/TR and LN229/TR cells to maintain their TMZ resistance. Analysis of the expression of HOTAIR in the TMZ-resistant GSE100736 data set showed that HOTAIR was highly expressed in the TMZ-resistant GBM cell line (Fig. 2E). The results of the cell counting kit-8 (CCK-8) assay showed that the half-maximal inhibitory concentration (IC50) of U251 and U251/TR cells was 43.1 μmol/L and 189.5 μmol/L, respectively. In addition, the IC50 of LN229 and LN229/TR cells was 26.68 μmol/L and 112.5 μmol/L (Fig. 2F). RT-qPCR results suggested that TMZ-resistant cell lines U251/TR and LN229/TR exhibited higher expression of HOTAIR than the parental cells U251 and LN229 (Fig. 2G). Furthermore, colony formation assay results showed more colonies formed in the cells co-cultured with GBM-serum-EVs than the healthy-serum-EVs-treated cells (Fig. 2H), revealing that healthy-serum-EVs-treated cells were more sensitive to TMZ. Flow cytometric data suggested a decline of cell apoptosis upon treatment with GBM-serum-EVs compared with healthy-serum-EVs treatment (Fig. 2I). Taken together, these lines of evidence indicate that GBM-serum-EVs may stimulate malignant features as well as TMZ resistance of GBM cells by mediating HOTAIR.

### HOTAIR competitively binds to miR-526b-3p in U251 cells

**Fig. 3:**
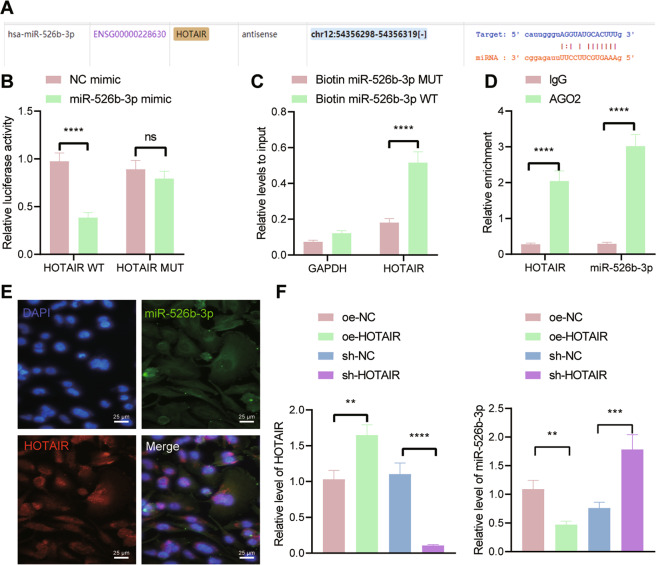
HOTAIR competitively binds to miR-526b-3p in U251 cells. **A** The predicted binding sites between HOTAIR and miR-526b-3p by the starBase database. **B** Binding between miR-526b-3p and HOTAIR confirmed by dual-luciferase reporter assay. **C** Enrichment of HOTAIR in U251 cells transfected with Bio-miR-526b-3p-WT or Bio-miR-526b-3p-MUT assessed by RNA pull-down assay. **D** Binding between HOTAIR and miR-526b-3p analyzed by RIP assay. **E** FISH analysis of subcellular localization of HOTAIR and miR-526b-3p in U251 cells. **F** RT-qPCR detection of miR-526b-3p expression in U251 cells treated oe-HOTAIR or sh-HOTAIR. **p < 0.01, ***p < 0.001 or ****p < 0.0001. ns indicates no significant difference between the two groups. Each experiment was repeated three times independently.

We then moved to identify the downstream factor of HOTAIR. The starBase database predicted that HOTAIR targeted miR-526b-3p (Fig. 3A). The results of the dual-luciferase reporter assay further revealed that the luciferase activity of HOTAIR-WT was reduced in U251 cells transfected with miR-526b-3p mimic, which showed no effect on the luciferase activity of HOTAIR-MUT (Fig. 3B). Moreover, the results of RNA pull-down assay indicated that miR-526b-3p-WT enriched more HOTAIR (Fig. 3C). RIP assay data showed that Ago2 antibody could co-immunoprecipitate both HOTAIR and miR-526b-3p (Fig. 3D). As shown in Fig. 3E, HOTAIR and miR-526b-3p were colocalized in the cytoplasm. Furthermore, the expression of miR-526b-3p was reduced in U251 cells overexpressing HOTAIR while it was augmented in the absence of HOTAIR (Fig. 3F). Overall, these findings support that HOTAIR can bind to miR-526b-3p in U251 cells.

### miR-526b-3p targets EVA1 and inhibits its expression in U251 cells

**Fig. 4:**
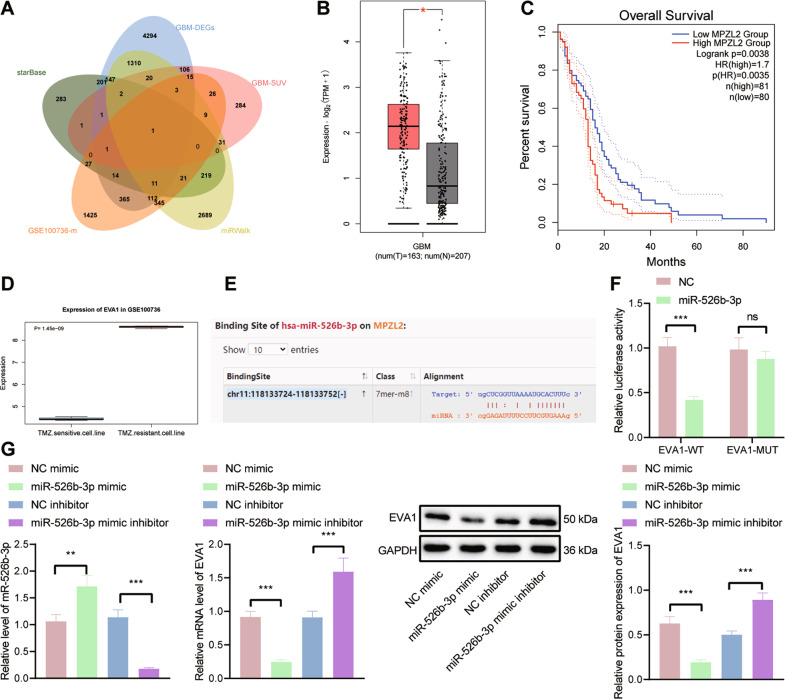
EVA1 is a target gene of miR-526b-3p. **A** Venn diagram of downstream genes of miR-526b-3p predicted by the starBase and miRWalk databases, the top 500 survival-related genes, and TMZ resistance-related genes from the GSE100736 data set. **B** A box plot of EVA1 expression in TCGA-GBM. The red box on the left indicates GBM samples, and the gray box on the right indicates normal samples. **C** Correlation analysis of EVA1 expression with the overall survival of GBM patients in TCGA-GBM. **D** EVA1 expression in the TMZ resistance-related GSE100736 data set. **E** The predicted binding sites between miR-526b-3p and EVA1 by the starBase database. **F** Binding of miR-526b-3p to EVA1 confirmed by dual-luciferase reporter assay. **G** RT-qPCR and western blot analysis detection of EVA1 expression in U251 cells transfected with miR-526b-3p mimic or miR-526b-3p inhibitor. *p < 0.05, **p < 0.01, or ***p < 0.001. ns indicates no significant difference between the two groups. Each experiment was repeated three times independently.

To determine the downstream genes of miR-526b-3p, we employed the starBase and miRWalk databases, which revealed 929 and 4920 downstream genes of miR-526b-3p, respectively. In addition, 2375 genes related to TMZ resistance in GBM were obtained from the GSE100736 data set. Intersection analysis of these genes and survival-related genes revealed that EVA1 was the most critical downstream gene (Fig. 4A). In the TCGA-GBM database, EVA1 was observed to be highly expressed in GBM and this high expression was linked to the poor prognosis of GBM patients (Fig. 4B, C). Analysis of the GSE100736 data set showed that EVA1 was upregulated in the TMZ-resistant GBM cell lines (Fig. 4D).

The starBase database predicted the presence of binding sites between miR-526b-3p and EVA1 (Fig. 4E). Dual-luciferase reporter assay data further confirmed their binding, as the luciferase activity of EVA1-WT was inhibited in U251 cells transfected with miR-526b-3p mimic while that of EVA1-MUT had no alterations (Fig. 4F). Moreover, RT-qPCR and western blot analysis results showed that the expression of EVA1 was reduced in U251 cells transfected with miR-526b-3p mimic but an opposite result was caused by miR-526b-3p inhibitor (Fig. 4G). Thus, it can be concluded that miR-526b-3p targets EVA1 and suppresses its expression in U251 cells.

### HOTAIR upregulates the expression of EVA1 by competitively binding to miR-526b-3p, thus promoting malignant phenotypes and TMZ resistance in GBM

**Fig. 5:**
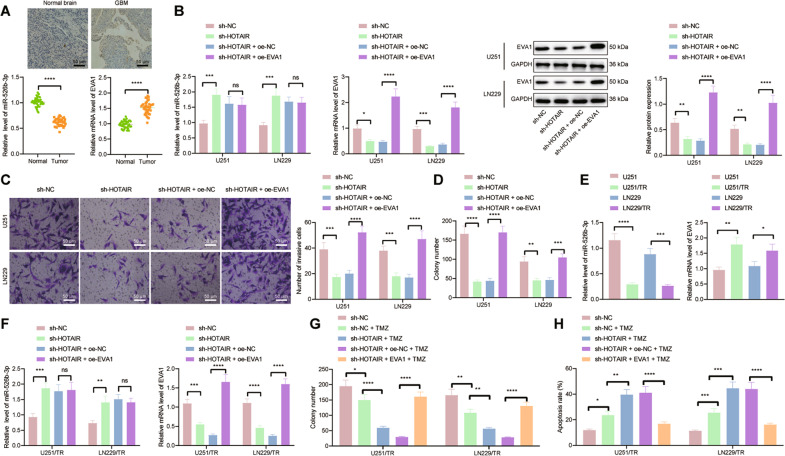
HOTAIR accelerates GBM progression and increases TMZ resistance by regulating the miR-526b-3p/EVA1 axis. **A** miR-526b-3p expression detected by RT-qPCR and EVA1 mRNA and positive expression detected by RT-qPCR and immunohistochemistry in clinical tissues of GBM patients (*n* = 40) and normal brain tissues (*n* = 40). *p < 0.05, compared with normal brain tissues. **B** miR-526b-3p expression detected by RT-qPCR and EVA1 mRNA and protein expression detected by RT-qPCR and western blot analysis in U251 and LN229 cells treated with sh-HOTAIR or combined with oe-EVA1. **C** Invasion of U251 and LN229 cells treated with sh-HOTAIR or combined with oe-EVA1 measured by Transwell assay. **D** Colony formation of U251 and LN229 cells treated with sh-HOTAIR or combined with oe-EVA1 measured by colony formation assay. **E** RT-qPCR detection of miR-526b-3p and EVA1 expression in U251/TR and LN229/TR cells as well as parental cells U251 and LN229. **F** RT-qPCR detection of miR-526b-3p and EVA1 expression in U251/TR and LN229/TR cells treated with sh-HOTAIR or combined with oe-EVA1. **G** Colony formation of U251/TR and LN229/TR cells treated with sh-HOTAIR or combined with oe-EVA1 measured by colony formation assay. **H** Flow cytometric analysis of U251/TR and LN229/TR cell apoptosis upon treatment with sh-HOTAIR or combined with oe-EVA1. *p < 0.05, **p < 0.01, ***p < 0.001, or ****p < 0.0001. ns indicates no significant difference between the two groups. Each experiment was repeated three times independently.

The following experiments were focused on exploring the molecular mechanism by which HOTAIR regulated the miR-526b-3p/EVA1 axis and affected GBM progression and TMZ resistance. The results of RT-qPCR and immunohistochemistry showed that miR-526b-3p was weakly expressed while EVA1 was highly expressed in clinical tissues of GBM patients (Fig. 5A). Besides, we observed an increase of miR-526b-3p level and a decline of EVA1 level in U251 and LN229 cells treated with sh-HOTAIR, whereas EVA1 level was augmented in the presence of sh-HOTAIR + oe-EVA1 (Fig. 5B). Furthermore, cell proliferative and invasive phenotypes were reduced in the absence of HOTAIR while combined treatment with sh-HOTAIR and oe-EVA1 led to opposite results (Fig. 5C, D). The above results indicate that HOTAIR binds to miR-526b-3p and upregulates EVA1, thus promoting the progression of GBM.

Meanwhile, the RT-qPCR results revealed lower miR-526b-3p level and higher EVA1 level in U251/TR and LN229/TR cells than in parental cells U251 and LN229 (Fig. 5E). In addition, miR-526b-3p level was augmented and EVA1 level was downregulated in U251/TR and LN229/TR cells treated with sh-HOTAIR, the effect of which was abolished by further EVA1 overexpression (Fig. 5F). The number of colonies formed in the sh-HOTAIR-treated cells was reduced but apoptosis was increased, which was negated following treatment with both sh-HOTAIR and oe-EVA1 (Fig. 5G, H). Therefore, HOTAIR may have the potential to elevate the expression of EVA1 by competitively binding to miR-526b-3p, consequently inducing GBM progression and TMZ resistance.

### GBM-serum-EVs containing HOTAIR facilitate tumor growth and TMZ resistance by regulating the miR-526b-3p/EVA1 axis in vivo

**Fig. 6:**
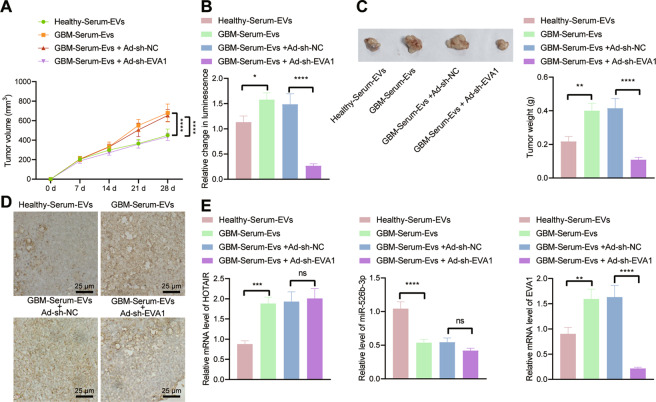
HOTAIR-loaded in serum-EVs increases tumor growth and TMZ resistance in vivo via the miR-526b-3p/EVA1 axis. **A** Tumor volume of mice injected with U251 cells co-cultured with GBM-serum-EVs or combined with Ad-sh-EVA1. **B** In vivo luminescence changes of mice injected with U251-LUC cells co-cultured with GBM-serum-EVs or combined with Ad-sh-EVA1. **C** Representative images showing xenografts and tumor weight analysis of mice injected with U251 cells co-cultured with GBM-serum-EVs or combined with Ad-sh-EVA1. **D** Immunohistochemistry analysis of Ki67 protein in tumor tissues of mice injected with U251 cells co-cultured with GBM-serum-EVs or combined with Ad-sh-EVA1. **E** RT-qPCR detection of HOTAIR, miR-526b-3p, and EVA1 expression in tumor tissues of mice injected with U251 cells co-cultured with GBM-serum-EVs or combined with Ad-sh-EVA1. *p < 0.05, **p < 0.01, ***p < 0.001 or ****p < 0.0001. ns indicates no significant difference between the two groups. Each experiment was repeated three times independently. *n* = 10 for mice following each treatment.

Finally, we sought to identify the role of serum-EVs containing HOTAIR in tumor resistance to TMZ by regulating the miR-526b-3p/EVA1 axis in vivo. U251-LUC cells were injected into the right brain of mice, which were then intraperitoneally injected with luciferase substrates (150 mg/kg) every 5 days for the monitoring of xenografts (the luminescence intensity was detected 10–15 min after each injection using in vivo imaging). When the luminescence intensity reached 3.0 × 10^6^ p/s/cm^2^/Sr, the mice were injected with TMZ. As depicted in Fig. 6A–D, treatment with GBM-serum-EVs led to increased tumor volume and weight as well as Ki67 protein expression than healthy-serum-EVs treatment. In contrast, further silencing of EVA1 caused opposite results. RT-qPCR results illustrated higher expression of HOTAIR and EVA1 and lower miR-526b-3p level in tumor tissues of mice injected with U251 cells co-cultured with GBM-serum-EVs. EVA1 level was found to be diminished upon dual treatment with GBM-serum-EVs + Ad-sh-EVA1 (Fig. 6E). As the HOTAIR level in serum-derived was unable to be directly knocked down, we knocked down in vivo HOTAIR expression through injection of adenovirus carrying sh-HOTAIR (Ad-sh-EVA1). The combination of GBM-Serum-EVs with Ad-sh-EVA1 was shown to reverse the promoting effect of GBM-Serum-EVs alone on TMZ resistance in GBM xenografts (Supplementary Fig. 1A–C). These lines of evidence demonstrate that HOTAIR-loaded on GBM-serum-EVs advances TMZ resistance in vivo through the miR-526b-3p/EVA1 axis.

## Discussion

GBM is a highly aggressive form of brain tumor and TMZ chemotherapy can significantly improve survival in GBM patients, many of whom, however, do not respond to TMZ or exhibit resistance [13]. Therefore, attempts should be made to understand TMZ resistance mechanisms and to develop effective treatments to maximize patient benefit. Our work supported the promoting effect of GBM-serum-EVs on the proliferative and invasive phenotypes and TMZ resistance of GBM cells by regulating the HOTAIR/miR-526b-3p/EVA1 axis.

Our initial results provided evidence suggesting that HOTAIR was highly expressed in clinical tissues, cells, and serum-EVs of GBM. Consistently, HOTAIR has been detected to be overexpressed in clinical tissue samples from patients with GBM [14]. Meanwhile, a previous study demonstrated enriched HOTAIR in the serum sample-derived EVs of GBM patients [15]. The subsequent findings in our work demonstrated that GBM-serum-EVs could augment GBM cell malignant features as well as induce TMZ resistance by carrying HOTAIR. EVs can encapsulate lncRNAs and deliver them to the recipient tumor cells, where lncRNAs exert promoting effects on tumor progression and chemoresistance [16]. Recent analysis has indicated that serum exosomal HOTAIR induces TMZ resistance in vitro and in vivo while its downregulation impedes malignant features of TMZ-resistant GBM cells [8]. In addition, loss of HOTAIR in GBM cells suppresses HK2 expression and leads to the ensuing suppression of proliferative phenotypes and enhancement of sensitivity to TMZ in vitro and in vivo [17] Overall, these data demonstrate that serum-EV HOTAIR can be used as a novel prognostic and diagnostic biomarker for GBM.

Several miRNAs have been confirmed to be targets of HOTAIR and have a negative correlation with HOTAIR, such as miR-122 and miR-20a-5p [18, 19]. Our study provided the first evidence that HOTAIR could bind to miR-526b-3p and reduced its expression in GBM cells. In line with our results, a previous study has revealed that miR-526b-3p is downregulated in glioma cells, and in contrast, its upregulation results in inhibited proliferative phenotypes and augmented apoptosis of glioma cells in vitro along with arrested tumor growth in vivo [20]. Thus, disruption of HOTAIR-mediated miR-526b-3p inhibition may serve as a suppressor to delay the progression of GBM.

Furthermore, the biological prediction website and luciferase reporter assay identified that miR-526b-3p targeted EVA1 and inhibited its expression in GBM cells. This represents the first evidence for the post-transcriptional regulation of EVA1 by miR-526b-3p in GBM cells and may have importance in regulating GBM progression. Notably, EVA1 expresses highly in GBM-initiating cells (tumorigenic cells resistant to radio- and chemotherapies) and in stem cell marker-expressing cells from GBM tissues, and EVA1 deficiency weakens GBM-initiating cell self-renewal potentials [12]. Mechanistic investigations in this study indicated that HOTAIR upregulated the expression of EVA1 by competitively binding to miR-526b-3p, thus enhancing GBM progression and TMZ resistance. Indeed, published data have confirmed that lncRNAs can act as miRNA sponges, and thus reduce their regulatory effects on the target mRNAs, thus accelerating the progression of glioma in vitro and in vivo [21]. Thus, targeting the HOTAIR/miR-526b-3p/EVA1 signaling might be a novel insight for GBM treatment.

Of note, the increase in TMZ resistance is a well-recognized contributor to the failure in GBM treatment [22]. Thus, molecular mechanisms underlying TMZ resistance in GBM have been extensively investigated, and the main findings to date fall into four categories. The intrinsic potential of glioma stem cells to survive, evolve, and repopulate the GBM tumor is the first to be implicated in TMZ resistance [23, 24]; second, the aberrant regulation of DNA mismatch repair (MMR) proteins is correlated with TMZ resistance in a fraction of GBM cases [25, 26]; some miRNAs (such as miR-195 and miR-455-3p) are also involved in the acquisition of TMZ resistance, affecting cell-killing effect in GBM in the presence of TMZ [27]; on the other hand, GBM tumor cell-derived EVs were reported to potentiate TMZ resistance in GBM, possibly by transferring related miRNA or harboring ZM fusion [28, 29]. In spite of the aforementioned extensive investigations regarding TMZ resistance and several ones that have indicated the TMZ resistance-promoting role of HOTAIR [19], the present study stands out for exploring the clinically isolated EVs in both in vitro and in vivo models, wherein HOTAIR-loaded GBM-serum-EVs were reasonably elucidated to be a novel prognostic and diagnostic biomarker for GBM.

**Fig. 7:**
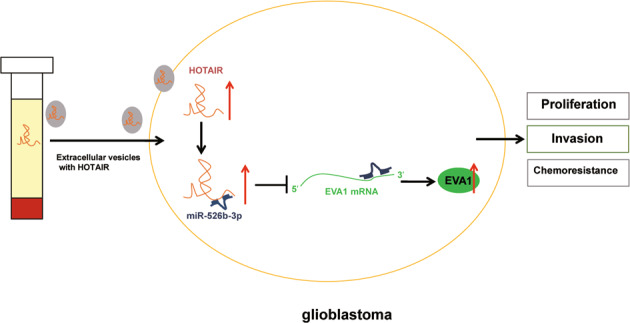
Schematic diagram of the mechanism by which GBM-serum-EVs affect GBM cell proliferation, invasion, and chemoresistance. GBM-serum-EVs deliver HOTAIR to GBM cells, where HOTAIR binds to miR-526b-3p and upregulates the expression of its target gene EVA1, thereby stimulating the proliferation, invasion, and chemoresistance of GBM cells.

Overall, our study indicates that serum-EVs deliver HOTAIR to GBM cells where HOTAIR binds to miR-526b-3p and upregulates the expression of EVA1, thereby stimulating the GBM progression and TMZ resistance (Fig. 7). Elaboration on the HOTAIR/miR-526b-3p/EVA1 pathway may provide a better understanding of chemoresistance in GBM, and new targets for the prevention and treatment of GBM. Nonetheless, the interaction of the HOTAIR/miR-526b-3p/EVA1 warrants further investigation owing to the little supporting literature elucidating the targeting between them.

## Materials and methods

### Bioinformatics analysis

The GBM-related data set in TCGA was retrieved from the GEPIA database. Significantly upregulated genes were screened with logFC >0.8 and *p* < 0.05 set as the threshold. GEO2R tool was used for differential analysis on the TMZ resistance-related data set GSE100736 (six samples consisted of three cases of TMZ-resistant cell lines and three cases of TMZ-sensitive cell lines) from the GEO database. The top 500 genes related to GBM survival were identified following GEPIA analysis. Human lncRNAs were obtained from the GENCODE database. Downstream miRNAs of lncRNAs were predicted using the starBase database while miRNA downstream genes used the starBase and miRWalk databases.

### Sample collection

GBM tissues were surgically obtained from 40 patients (22 males and 18 females; aged 23–76 years with a mean age of 47 years) with GBM at Huizhou Third People’s Hospital, Guangzhou Medical University from June 2018 to June 2020. None of the patients with GBM received antitumor treatment before sample collection. Besides, 40 cases of normal brain tissues were collected from patients experiencing surgeries due to trauma, epilepsy, or vascular malformations. The tissue samples were stored at −80 °C. Further, the serum was harvested from GBM patients and healthy donors to isolate EVs.

### Isolation and identification of serum-EVs

ExoQuick kit (EXOQ5TM-1-SBI, System Biosciences, Inc., Mountain View, CA) was applied to isolate EVs from serum. In brief, the serum samples of patients with GBM were taken out and thawed on ice. Next, 400 μL of serum samples were drawn, added with 100 μL of ExoQuick reagent, mixed, and left to stand for 30 min at 4 °C. The samples were centrifuged at 1500 × *g* for 30 min, and the supernatant was discarded. Additional centrifugation was conducted at 1500 × *g* for 5 min, and the resulting precipitate was EVs. Particles containing EVs were suspended in PBS for EV characterization and RNA sample extraction.

The morphology of the isolated EVs was then observed under a transmission electron microscope. Nanoparticle tracking analysis (NTA) was performed to measure the size distribution of EVs. Briefly, the EV sample was diluted with 0.15 M NaCl at a ratio of 1: 50, mixed, and subjected to NTA utilizing a Zetasizer Nano-ZS90 instrument (Malvern Instruments, Malvern, UK) at an excitation wavelength of 532 nm. The experiment was performed in triplicates.

The EVs were lysed with the radioimmunoprecipitation assay (RIPA) lysis buffer supplemented with protease inhibitor, followed by the determination of protein concentration utilizing the BCA protein assay kit (A53226, Thermo Fisher Scientific, Rockford). The expression of EV-specific markers (CD63, CD9, TSG101, and GM130) was detected using western blot analysis to identify the characteristics of EVs.

### Cell culture and treatment

HEK293T cell line and GBM cell lines U251, LN229, LN18, and T98G were purchased from American Type Culture Collection (Manassas, VA), and human primary astrocyte NHA from Cell Bank of the Chinese Academy of Sciences (Shanghai, China). Cells were cultured in Dulbecco’s modified Eagle’s medium (DMEM; 10569044, Gibco, Grand Island, NY) with 10% FBS, 2 mM l-glutamine (Sigma-Aldrich, St Louis, MO), 100 U/mL penicillin, and 100 μg/mL streptomycin in a 5% CO_2_ incubator at 37 °C.

Cells were transfected with overexpression-negative control (oe-NC), oe-HOTAIR, sh-NC, short hairpin RNA (sh)-HOTAIR, mimic-NC, miR-526b-3p mimic, inhibitor-NC, and miR-526b-3p inhibitor using the Lipofectamine 2000 transfection reagent (11668-019, Invitrogen Inc., Carlsbad, CA). The transfection sequence and plasmid were purchased from Shanghai GenePharma Co, Ltd. (Shanghai, China).

The firefly luciferase Luc2P was cloned into the multiple cloning sites of the overexpression plasmid pHAGE-CMV-MCS-IRES-ZsGreen and the shRNA plasmid pSuper-retro-puro to construct the pHAGE-puro-Luc and pSuper-retro-puro-Luc vectors. The pHAGE-puro-Luc series plasmids and adjuvant plasmids pSPAX2, pMD2.G, pSuper-retro-puro-Luc series plasmids, and adjuvant plasmids gag/pol and VSVG were co-transfected into 293 T cells and cultured for 48 h. The GBM cells were transduced with lentivirus carrying sh-NC and sh-EVA1. After 48 h of infection, the GFP expression efficiency was assessed under a fluorescence microscope. shRNA sequences are shown in Supplementary Table 1.

### Construction of TMZ-resistant cell lines

TMZ-resistant cell lines (U251/TR and LN229/TR) were produced by their parental cells (U251 and LN229), and the initially sensitive U251 and LN229 cells were exposed to the culture solution with a gradual increase of TMZ concentration (final 400 μmol/L) for 6 months to screen GBM cells with TMZ resistance. During incubation, TMZ-resistant cell lines (U251/TR and LN229/TR) were added with 50 μmol/L TMZ to maintain resistance.

### CCK-8 method

Cell suspension (100 μL) was seeded (5 × 10^4^ cells/well) into a 96-well plate and cultured with 5% CO_2_ at 37 °C. After 24 h, cells were exposed to different concentrations of TMZ for 48 h. With the original culture medium discarded, cells continued to culture with serum-free DMEM (90 μL), and CCK-8 reagent (10 μL, K1018, Apexbio) with 5% CO_2_ at 37 °C for 2 h. The optical density value at 450 nm was measured.

### RNA isolation and quantitation

The total RNA was isolated from cells and tissues through TRIzol reagents (16096020, Thermo Fisher Scientific Inc., Waltham, MA) and then reverse-transcribed into complementary DNA (cDNA) using a Reverse Transcription kit (RR047A, TaKaRa, Tokyo, Japan, for mRNA detection) and Poly(A) Tail-Length Assay Kit (B532451, Sangon, Shanghai, China, for miRNA detection). RT-qPCR was conducted using SYBR Premix Ex TaqTM (DRR081, TaKaRa) on an ABI 7500 instrument (Applied Biosystems, Foster City, CA). LncRNA and mRNA were normalized to GAPDH while miRNA to U6, respectively (Supplementary Table 2). Quantification was performed by the 2^−ΔΔCt^ method.

Further, EVs were isolated from the medium used for the same volume of serum, followed by the extraction of EV-packaged RNA utilizing the Seramir EXOSME RNA PURIFICATION for Media & Urine kit (RA806TC-1, System Biosciences). For qRT-PCR assay, extracted lncRNAs were normalized to synthesized exogenous reference λ polya + RNA50 using the External Standard Kit (3789, Takara). In brief, the EV suspension was added with 1.8 × 10^8^ λ Polya + RNA; RNA was extracted utilizing the Seramir kit and subjected to RT-PCR detection with a 10-μl reaction system, followed by addition of 1-μl cDNA and then the qRT-PCR assay based on protocols of the Sybr Premix EX TAQ kit (Takara).

### Western blot analysis

After cell transfection and treatment, total protein was isolated from cells with RIPA lysis buffer (P0013B, Beyotime, Shanghai, China) supplemented with 1% protease inhibitor and phosphorylase inhibitor, followed by the protein concentration determination utilizing a bicinchoninic acid kit (A53226, Thermo Fisher Scientific). The protein sample (30 μg) was mixed with the same volume of loading buffer and boiled for 5 min before the protein separation using 10% sodium dodecyl sulfate–polyacrylamide gel electrophoresis. Samples were then transferred onto polyvinylidene difluoride membranes through wet transfer. The membranes were blocked using 5% bovine serum albumin (BSA) at room temperature for 1 h and probed overnight at 4 °C with the diluted primary antibodies EVA1 (bs-11080R-1, 1:500, Bioss, Beijing, China), CD9 (ab223052, 1:500, Abcam), TSG101 (ab30871, 1:1000, Abcam), and GAPDH (ab9485, 1:2500, Abcam). The next day, the membranes were incubated with horseradish peroxidase-labeled secondary antibody IgG (ab6721, 1:5000, Abcam). The immunocomplexes were visualized utilizing ECL reagent and band intensities were quantified using ImageJ 1.48 u software (NIH, Bethesda, Maryland), with GAPDH serving as a loading control.

### EV internalization

For labeling, EVs were incubated with 0.1 μM PKH67 (MIDI26, Sigma-Aldrich) for 10 min. The sample was then centrifuged with EXOSOME Spin Column MW (Invitrogen) to remove unbound dyes, followed by counting in NTA using the NanoSight NS500 instrument. The PKH67-labeled EVs (5.75 × 10^9^ particles) were subsequently added to corresponding GBM cells for 24-h co-incubation, after which fluorescence observation was conducted. Besides, the cells had been treated for 30 min with Latrunculin A (1 ng/mL), an inhibitor of endocytosis [30], before the co-incubation with EVs.

### RNA pull-down assay

Cells overexpressing HOTAIR were transfected for 48 h with 50 nM biotinylated miR-526b-3p (Bio-miR-526b-3p-WT), and biotinylated miR-526b-3p-MUT (Bio-miR-526b-3p-MUT). The cell lysate was incubated with M-280 streptavidin magnetic beads (S3762, Sigma-Aldrich) pre-coated with RNase-free BSA and yeast tRNA (Sigma-Aldrich) at 4 °C for 3 h. The cell lysate was rinsed twice with pre-cooled lysis buffer, three times with low salt buffer, and once with high-salt buffer. The bounded RNA was purified using TRIzol reagent and the enrichment of HOTAIR was examined by RT-qPCR.

### RNA binding protein immunoprecipitation (RIP)

RIP kit (#17-700, Millipore) was applied to assay the binding between HOTAIR and miR-526b-3p. Cells were centrifuged and the supernatant was harvested, a portion of which was removed as input, and the other was probed with antibodies against rabbit anti-human Ago2 (2 μg, ab32381, Abcam) and rabbit anti-human IgG (2 μg, ab109489, Abcam, taken as NC) for co-precipitation. The sample and input were digested with proteinase K and immunoprecipitated RNA was isolated and quantified by RT-qPCR.

### Fluorescence in situ hybridization (FISH)

Subcellular localization of HOTAIR and miR-526b-3p was determined using FISH as per the instructions of the Ribo^TM^ lncRNA FISH Probe Mix (Red) (Ribo Biotechnology, China) and miRNA FISH Probe (Bes5313, BersinBio, Guangdong, China). In brief, cells were fixed at room temperature with 4% paraformaldehyde (1 mL). Following treatment with proteinase K (2 μg/mL), glycine, and acetamidine reagent, cells were added with 250 μL prehybridization solution and incubated at 42 °C for 1 h. Then, cells were added with 250 μL hybridization solution containing the probe (300 ng/mL), and hybridized overnight at 42 °C. After the cells were rinsed thrice with PBST, DAPI (1:800) dye solution diluted with PBST was added to the 24-well plate to stain the nucleus for 5 min. At last, five different visual fields were randomly selected under a laser scanning confocal microscope (Leica, Wetzlar, Germany) for observation and photograph.

### Dual-luciferase reporter assay

The full length of HOTAIR was inserted into the dual-luciferase reporter gene vector pmirGLO (3577193, Promega Corporation, Madison, WI). U251 cells were seeded in 24-well plates and 24 h later, the mimic-NC and miR-526b-3p mimic were co-transfected with pmirGLO-HOTAIR-WT and pmirGLO-HOTAIR-MUT into U251 cells using Lipofectamine 2000 reagent (Invitrogen). The luciferase activity, normalized to renilla luciferase activity, was examined with the Dual-Luciferase Reporter Assay System (E1910, Promega). The sequences of HOTAIR-WT and HOTAIR-MUT are listed in Supplementary Table 3.

### Colony formation assay

Cells were seeded in a six-well plate at a density of 1 × 10^3^ cells/well and cultured in a 5% CO_2_ incubator at 37 °C for 10–15 days, with the medium changed every 4–5 days. Following supernatant removal, cells were fixed with 5 mL 4% paraformaldehyde and stained with 0.5% crystal violet. The number of cell colonies was observed under a microscope.

### Transwell assay

A Transwell chamber (pore size of 8 μm) in 24-well plates was used for this experiment. For cell invasion measurement, 100 μL of Matrigel was spread in each chamber and incubated at 37 °C for 2 h. Each chamber was added with 200 μL cell suspension, and 700 μL complete medium with 10% fetal bovine serum (FBS) was supplemented to the lower chamber, followed by incubation with 5% CO_2_ at 37 °C. After 48 h, the cells were fixed with methanol and stained with 0.5% crystal violet. The invaded cells were counted and photographed under an inverted optical microscope (Olympus).

### Flow cytometry

Annexin-V-FITC/PI double staining method was applied to assay cell apoptosis. After 48 h of transfection, cells were trypsinized and centrifuged at 800 × *g*. Based on the protocols of BD Annexin-V-FITC Apoptosis Detection Kit Ι, cells were resuspended in 500 μL binding buffer and incubated with 5 μL Annexin-V-FITC and 5 μL PI. Finally, cell apoptosis was measured on flow cytometry (FACSCalibur; BD, San Jose, CA).

### Immunohistochemistry

GBM tissue sections were quenched in 3% hydrogen peroxide. The slides were heated in 10 mM sodium citrate (pH 6.0) for 30 min, blocked with 10% normal goat serum for 15 min, and immunostained with primary antibody against Ki67 (ab15580, 1:1000, Abcam) at 4 °C overnight. After this, the sections were incubated with the secondary antibody goat anti-rabbit IgG (ab6721, 1:5000, Abcam) for 30 min. The sections were incubated with streptavidin–biotin peroxidase complex (Vector Labs (Burlingame, CA) in a 37 °C incubator for 30 min. Following development with DAPI (P0203, Beyotime), the sections were stained with hematoxylin for observation under an upright microscope (Olympus).

### In vivo luminescence imaging in nude mice

Four-week-old female BALB/c nude mice (Vital River Laboratory Animal Technology, Beijing, China) were housed in a specific pathogen-free environment with 60–65% humidity, at 22–25 °C, and under a 12-h light/dark cycle, with free access to food and water. The experiment was conducted after 1 week of acclimatization. The mice were assigned into four groups: healthy-serum-EVs (mice were injected with U251-LUC cells, i.e., luciferase-labeled U251 cells, that had been co-cultured with serum-EVs of healthy controls), GBM-serum-EVs (mice were injected with U251-LUC cells co-cultured with serum-EVs of GBM patients), GBM-serum-EVs + Ad-sh-NC (mice were injected with U251-LUC cells co-cultured with serum-EVs of GBM patients and transduced with adenovirus carrying sh-NC) and serum-EVs + Ad-sh-EVA1 (mice were injected with U251-LUC cells co-cultured with serum-EVs of GBM patients and transduced with adenovirus carrying Ad-sh-EVA1) (*n* = 10 for mice upon each treatment). Approximately 5 × 10^6^ U251-LUC cells were stereotactically injected into the right brain of mice (before orthotopic transplantation, U251-LUC cells were pre-co-cultured with designated EVs for 6 days). The situation of mice and tumor inoculation sites were observed every day, and intraperitoneal injection of luciferase substrate (150 mg/kg) into mice was conducted every 5 days. Tumor luminescence value was measured after 10–15 min. Imaging was performed using an in vivo animal imager (UVP iBox® Scientia™, Germany), with luminescence value calculated. When luminescence value reached 3.0 × 10^6^ p/s/cm^2^/sr, the mice were subjected to intraperitoneal injection of TMZ at a dose of 60 mg/kg/d every 5 days. Four weeks after inoculation, tumor luminescence value was determined using the same method. The mice were euthanized by intraperitoneal injection of 80 mg/kg of pentobarbital sodium, and the tumor tissue was removed for subsequent experiments.

### Statistical analysis

Statistical analysis was performed using GraphPad Prism 5 (GraphPad Software, La Jolla, CA). All experiments were conducted three times independently. The measurement data were described as mean ± standard deviation. Data between the two groups were compared using an unpaired *t* test. Data among multiple groups were analyzed by one-way analysis of variance. Pearson’s correlation coefficient was used for correlation analysis between indicators. A value of *p* < 0.05 was statistically significant.

## Supplementary information

Supplementary Figure 1

Supplementary Tables

Original Western Blots

aj-checklist

## Data Availability

The data sets generated/analyzed during our work are available.
