# Supplementary material for: Serum-derived extracellular vesicles facilitate temozolomide resistance in glioblastoma through a HOTAIR-dependent mechanism
Source: Cell Death Dis. 2022 Apr 13;13(4):344. doi: 10.1038/s41419-022-04699-8 (PMC9008004; doi:10.1038/s41419-022-04699-8)
Supplement: Supplementary file 1 — Supplementary Figure 1 [file 41419_2022_4699_MOESM1_ESM.docx]

**
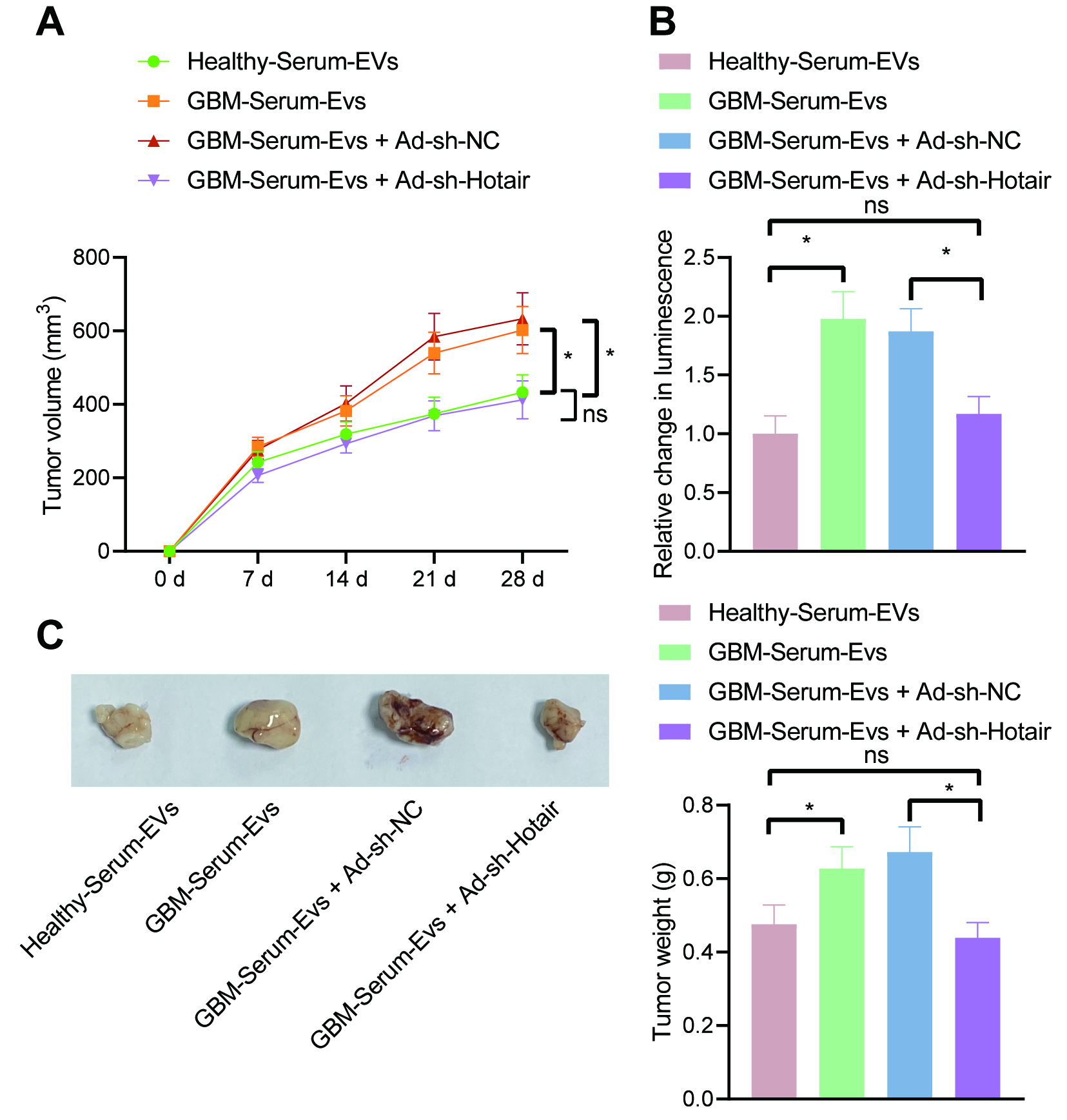
**

**Supplementary Fig. 1** GBM-Serum-derived EVs promote the TMZ resistance of GBM xenografts by delivering HOTAIR. A, Growth curve of xenografted GBM tumors in mice injected with GBM-Serum-EVs alone or in combination with adenovirus carrying sh-HOTAIR (Ad-sh-HOTAIR). B, Luciferase activity detected by *in vivo* luminescence imaging in mice injected with GBM-Serum-EVs alone or in combination with Ad-sh-HOTAIR. C, Representative images of GBM xenografts in mice injected with GBM-Serum-EVs alone or in combination with Ad-sh-HOTAIR. 10 mice per group. * *p* <0.05. n = 10 for mice following each treatment.
