# Supplementary material for: Serum-derived extracellular vesicles facilitate temozolomide resistance in glioblastoma through a HOTAIR-dependent mechanism
Source: Cell Death Dis. 2022 Apr 13;13(4):344. doi: 10.1038/s41419-022-04699-8 (PMC9008004; doi:10.1038/s41419-022-04699-8)
Supplement: Supplementary file 2 — Supplementary Tables [file 41419_2022_4699_MOESM2_ESM.docx]

**Supplementary Table 1** shRNA sequences

| shRNA | Sequence |
| --- | --- |
| sh-NC | 5’-CCTAAGGTTAAGTCGCCCTCG-3’ |
| sh-EVA1 | 5’-GTTTAACTCGAGTTAAACCGC-3’ |

**Supplementary Table 2** Primer sequences for reverse transcription quantitative polymerase chain reaction

| Gene | Primer sequence |
| --- | --- |
| HOTAIR | Forward: 5’-GGTAGAAAAAGCAACCACGAAGC-3’ |
|  | Reverse: 5’-ACATAAACCTCTGTCTGTGAGTGCC-3’ |
| miR-526b-3p | Forward: 5’-GCGCTCTTGAGGGAAGCACT-3’ |
|  | Reverse: Universal primer |
| U6 | Forward: 5’-CTCGCTTCGGCAGCACA-3’ |
|  | Reverse: Universal primer |
| EVA1 (MPZL2) | Forward: 5’-GAAAAAGCGATGGGCCGAAA-3’ |
|  | Reverse: 5’-TGGAAATCATCTCAGCTTCCATCT-3’ |
| GAPDH | Forward: 5’-AGAAGGCTGGGGCTCATTTG-3’ |
|  | Reverse: 5’-AGGGGCCATCCACAGTCTTC-3’ |

**Supplementary Table 3** Sequences of HOTAIR-WT and HOTAIR-MUT

| Plasmid | Sequence |
| --- | --- |
| HOTAIR-WT | 5’-CAUUGGGUAGGUAUGCACUUUG-3’ |
| HOTAIR-MUT | 5’-CAUUGGGUCAAGGCAUCUGGCA-3’ |
