# Supplementary material for: Serum-derived extracellular vesicles facilitate temozolomide resistance in glioblastoma through a HOTAIR-dependent mechanism
Source: Cell Death Dis. 2022 Apr 13;13(4):344. doi: 10.1038/s41419-022-04699-8 (PMC9008004; doi:10.1038/s41419-022-04699-8)
Supplement: Supplementary file 3 — Original Western Blots [file 41419_2022_4699_MOESM3_ESM.docx]

**Original Western Blots**

**Figure 1H**


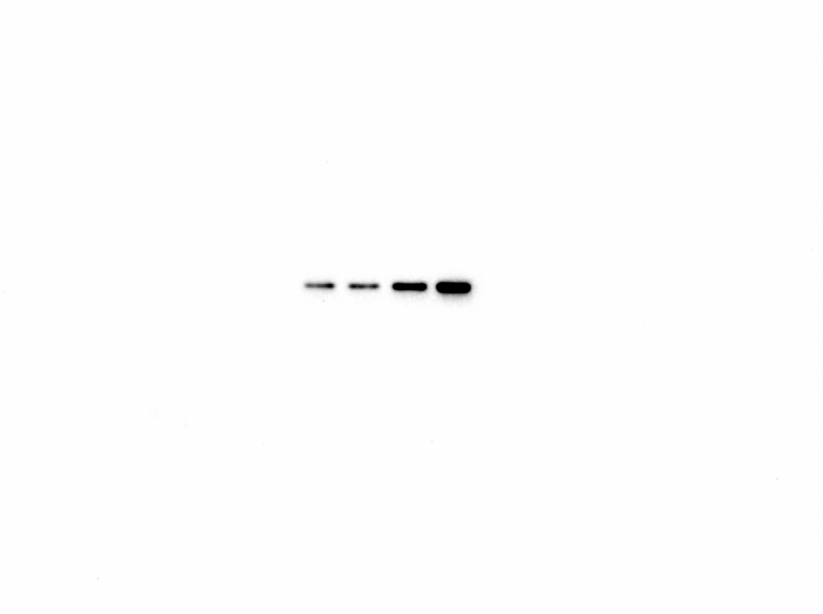
CD63


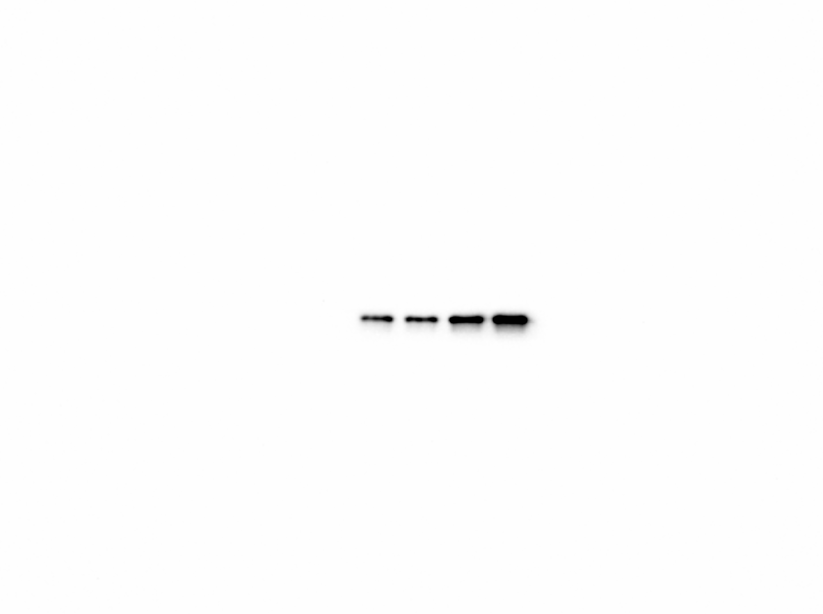
CD9


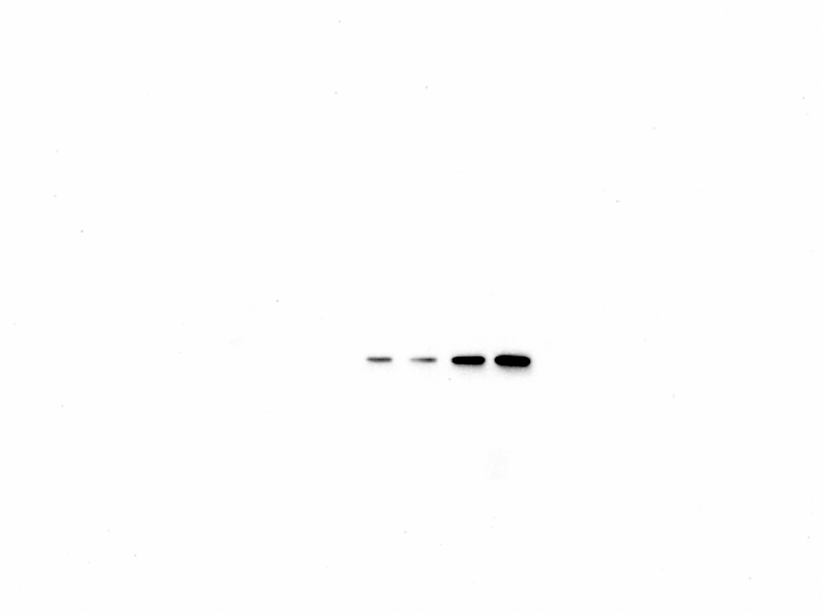
TSG101


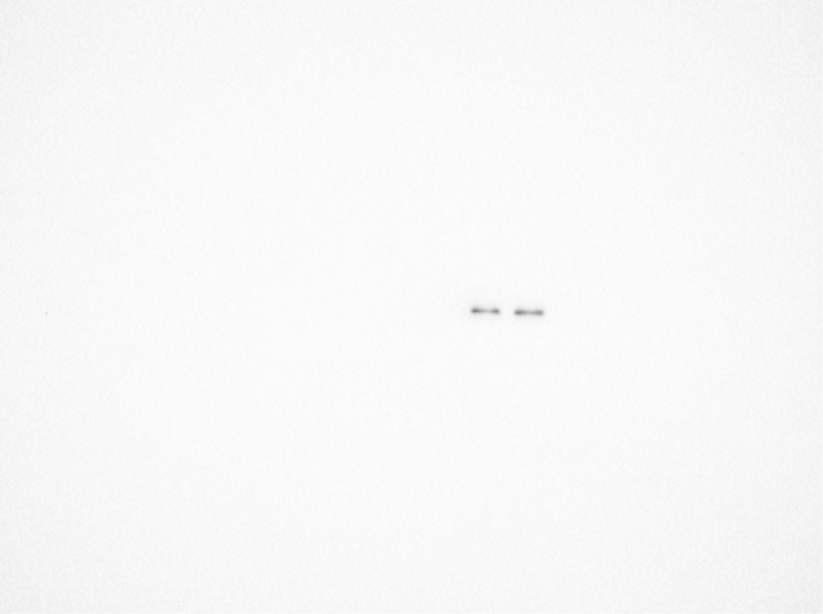
GM130


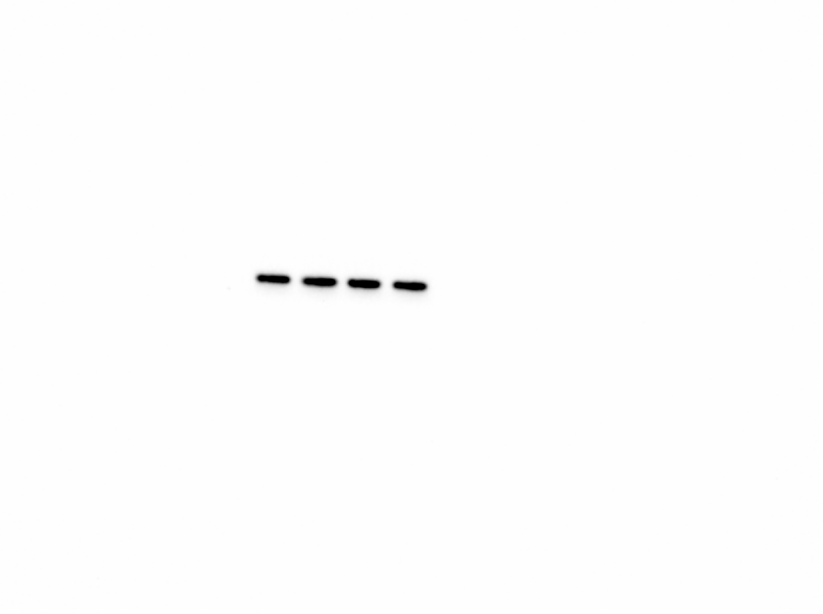
GAPDH

**Figure 4G**


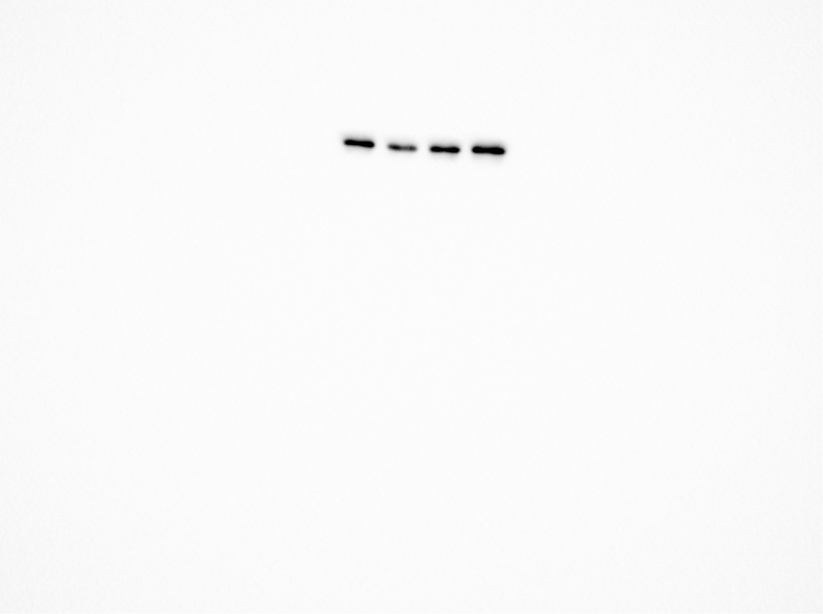
EVA1


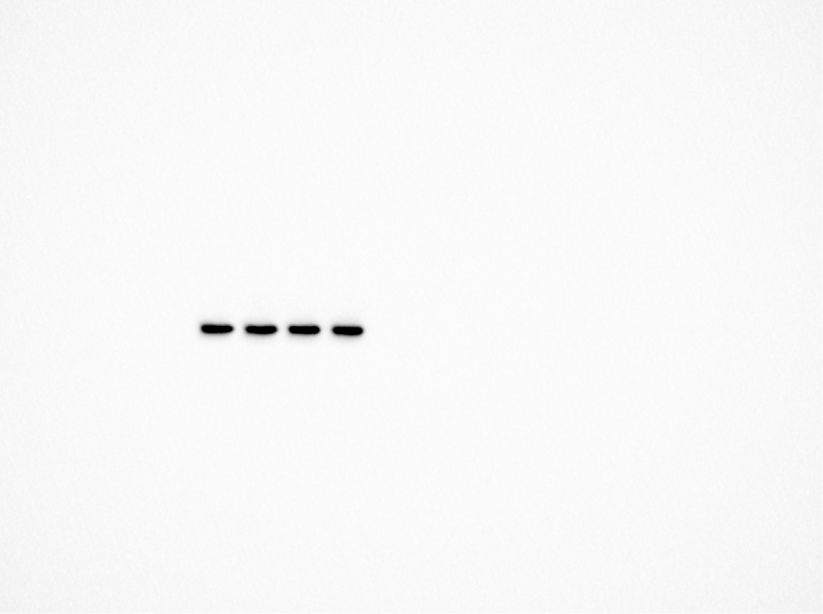
GAPDH

**Figure 5B**


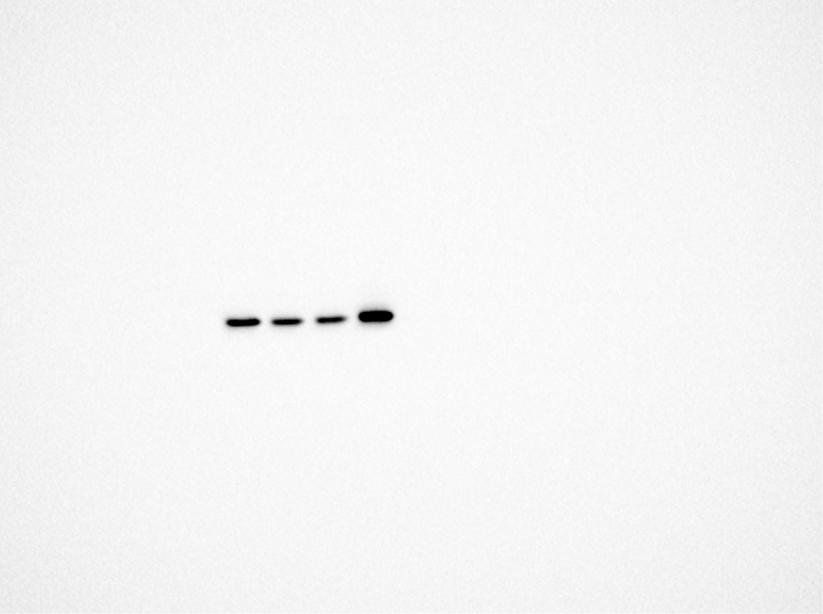
U251-EVA1


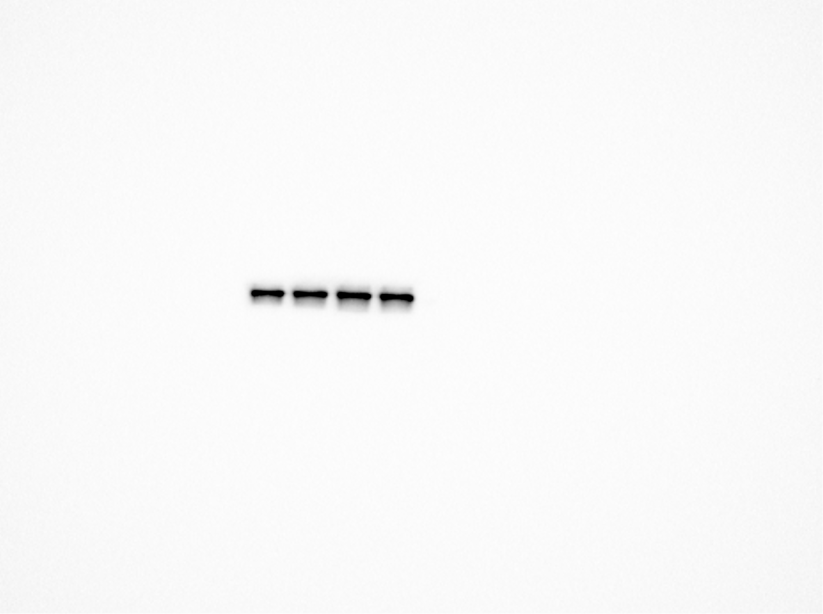
U251-GAPDH


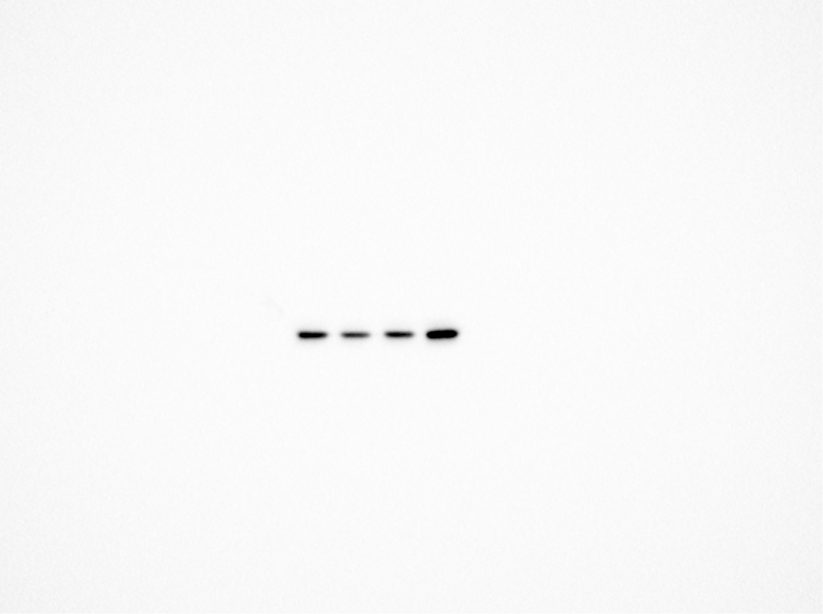
LN229-EVA1


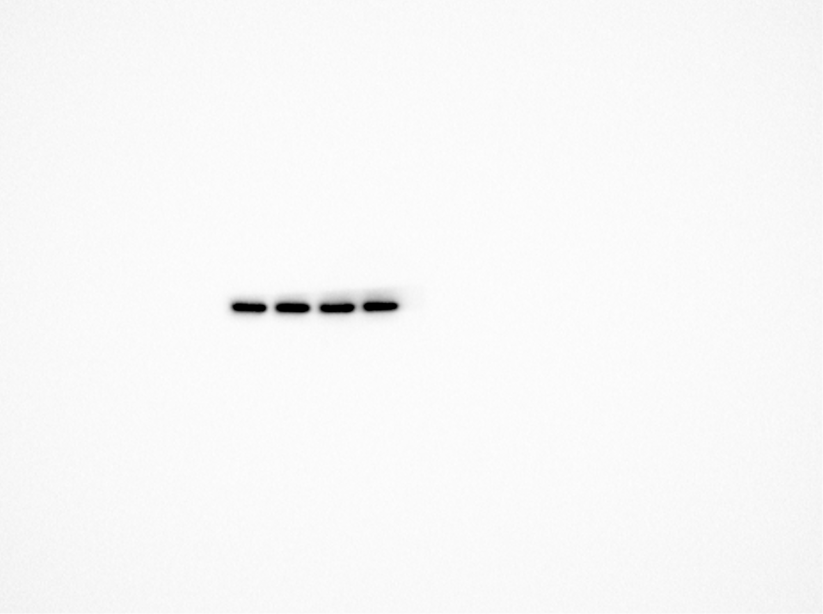
LN229-GAPDH
